# Antibiotic prescription pattern among Primary Healthcare General Practitioners in the South Batinah Governorate of Oman, 2019

**DOI:** 10.1186/s12875-024-02488-0

**Published:** 2024-08-10

**Authors:** Sami M. Al Mujaini, Zayid K. Almayahi, Noura A. Abouammoh, Sumaiya Al Amri

**Affiliations:** 1 Disease Surveillance and Control Department, Ministry of Health, South Batinah Governorate, P.O. Box: 131, P.C: 300, Rustaq, Oman; 2 U.S. Centers for Disease Control and Prevention, Middle East/North Africa (MENA) Regional Office, Muscat, Oman; 3 Department of Family and Community Medicine, College of Medicine, King Saud University, Riyad, Saudi Arabia; 4 Disease Surveillance and Control Department, Ministry of Health, Muscat, Oman

**Keywords:** Antimicrobial resistance, Primary health care, Antibiotics, Inappropriate prescriptions

## Abstract

**Background:**

Misuse and overuse of antibiotics comprise leading causes of antimicrobial resistance. The study aims to assess the pattern of antibiotic prescription among primary healthcare general practitioners in the South Batinah Governorate of Oman.

**Method:**

A cross-sectional study of 600 antibiotic prescriptions issued in the South Batinah Governorate in 2019 was conducted to verify the triggering diagnoses and determine the appropriateness of the prescribed antibiotic. Logistic regression analysis was used to determine the association between predictors and inappropriate use.

**Results:**

Respiratory infections accounted for 62% of antibiotic prescriptions, of which 92.2% were inappropriately prescribed. Extended-spectrum antibiotics were inappropriately prescribed in 33.3% of cystitis cases, while 14.3% of gastroenteritis received incorrect spectrum of antibiotics. Amoxicillin represented 46.2% of antibiotic prescriptions, of which 84.4% were unnecessarily prescribed. Lower inappropriate antibiotic prescribing rate was linked to patients ≥ 18 years (OR = 0.46, 95% CI: [0.26, 0.82]), those who underwent laboratory tests (OR = 0.22, 95% CI: [0.12, 0.39]), and consultations at health centers (OR = 0.44, 95% CI: [0.24, 0.79]). Arabic-speaking physicians were more likely to prescribe antibiotics inappropriately.

**Conclusion:**

Inappropriate antibiotic prescription was frequently observed in mild respiratory infections and associated with specific patient and physician characteristics. Appropriateness of antibiotic prescriptions issued can be improved through enhanced testing capacities as well as implementation of physician and community awareness campaigns.

**Supplementary Information:**

The online version contains supplementary material available at 10.1186/s12875-024-02488-0.

## Highlights

**•** Inappropriate prescriptions were common among respiratory symptoms.
**•** Amoxicillin was the most common antibiotic prescribed inappropriately.
**•** Laboratory–tested patients were less likely to receive inappropriate antibiotics.
**•** Inappropriate antibiotics were commonly prescribed by Arabic-speaking physicians.
**•** Health centers’ primary care physicians rarely prescribe inappropriate antibiotics.

**Supplementary Information:**

The online version contains supplementary material available at 10.1186/s12875-024-02488-0.

## Introduction

Antimicrobial resistance (AMR) is a rapidly rising global health concern, and is considered among the top ten public health risks [1] Death, disability and prolonged illnesses arising from AMR place a significant burden on national economies [1] Increased patient morbidity, and mortality rates can be caused by antibiotic misuse and overuse [1] Such practice arises from physicians’ failure to comply with antibiotic guidelines [1, 2] High levels of inappropriate antibiotic prescribing patterns have been noted among primary care physicians in different parts of the world [3, 4].

Globally, antibiotic prescriptions increased by 90.5% between 2000 and 2015 [5] In 2016, it was reported that in the United States, among all out-patient antibiotic prescriptions for privately insured children and non-elderly patients, 23.2% were inappropriate [5] A study of primary care physicians in China, which referenced national antibiotic prescription guidelines as well as those published by the US Centers for Disease Control and Prevention (CDC), observed that 91.8% of antibiotic prescriptions were inappropriately prescribed and that 70% were classified as unnecessary [3] Penicillin accounted for more than half of the antibiotic prescriptions; 84.4% of these scripts were prescribed unnecessarily [3].

Several studies have identified associations between the inappropriate prescription of antibiotics and patient and physician characteristics [6]. For instance, inappropriate prescribing was associated with male physicians, age less than 32 years, who had a lower level of postgraduate educational qualifications and doctors who had a high workload [3] Ineffective doctor-patient communication has been shown to increase the risk of inappropriate antibiotic prescription as a result of improper history taking and physical examination [7]. Antibiotic prescriptions are additionally subject to seasonal variation, with a higher frequency evident during the winter months [8].

A systematic review of the antibiotic prescribing patterns in out-patients and emergency departments in the Gulf Cooperation Countries (GCC) documented a high level of inappropriate antibiotic prescription reaching a maximum of 80% [5] This review also showed that respiratory tract infections, particularly upper respiratory infections, were the most common infection that led to the prescription of antibiotics; penicillins, such as amoxicillin and co-amoxiclav, and cephalosporins, e.g. ceftriaxone, were the most frequently prescribed [5].

Oman Antimicrobial Resistance Surveillance System was established in May 2016. The first report, published in 2018, showed an elevation of multi-drug resistant organisms from 91 per 1000 patients with bacteremia in 2017 to 98 per 1000 in 2018 [9] In addition, there was a high consumption of antibiotics per 1000 patient days noted, i.e. 396,482 and 431,460 defined daily doses (DDD) in 2017 and 2018, respectively [9]. In Oman, only two studies have been conducted to evaluate antibiotic prescribing practice. The first was carried out in adult in-patients at Sultan Qaboos University Hospital (SQUH), a tertiary hospital [10] The authors used SQUH antibiotic guidelines and showed that 63% of prescriptions failed to demonstrate guideline adherence. Piperacillin-tazobactam was the most common (31%) inappropriately prescribed antibiotic [10] The second study was conducted in SQUH in-patient and out-patient pediatric population, and reported that amoxicillin-clavulanate was the most commonly prescribed antibiotic in both in-patients (27.0%) and out-patients (33.9%) [11] To the best of our knowledge, there are no studies on whether or not antibiotic prescriptions are issued appropriately in the context of primary healthcare (PHC) institutions in Oman.

The aim of the current study is to evaluate the patterns of antibiotic prescription among general practitioners in governmental PHC general practitioners in the South Batinah Governorate (SBG) of Oman, and to examine the associated risk factors and trends over time.

## Methodology

### Study design

This is a cross-sectional study that analyzed antibiotic prescribing patterns and their appropriateness. Factors associated with inappropriate antibiotic prescribing practices are highlighted.

### Study setting

The study was conducted in the SBG of Oman in October 2022. The SBG is one of 11 governorates in Oman, and has 21 PHC facilities, 2 medical prevention centers and 1 secondary referral hospital. The SBG consists of 6 districts (wilayats) which have the following medical institutions: Rustaq (1 polyclinic, 2 local hospitals, 4 health centers), Al Awabi (1 local hospital, 2 health centers), Nakhal (1 local hospital, 2 health centers), Wadi Al Maawil (1 health center), Barka (1 polyclinic, 3 health centers) and Al-Musannah (1 polyclinic, 2 health centers). All the healthcare facilities run by the Ministry of Health (MoH) use the Al Shifa health information system. This is a comprehensive integrated medical information system designed to manage the medical, administrative, financial and legal operations of a hospital or a network of healthcare units as well as the corresponding processing services [12].

In Oman, general practitioners provide PHC services in three types of primary facilities; polyclinics (extended health centers), local hospitals and health centers [13] In addition to general PHC services, out-patient clinics dedicated to a range of specialties are available in polyclinics [13] Health centers and polyclinics provide out-patient services while local hospitals offer PHC facilities for the inhabitants of nearby villages as well as in-patient day care for those patients who need continuous monitoring [13]. Health centers and local hospitals have limited laboratory investigations, i.e., serum white cell count, and stool and urine analysis, which can be used to identify some bacterial infections. Thus, samples are sent to the polyclinics for further tests when required. There is no organism identification and antibiotic susceptibility tests capacity available in any of the PHC institutions. However, urine and stool specimens are sent to the regional hospital for cultures and susceptibility tests when recommended. Each PHC facility serves a specific catchment area; from administrative and technical perspectives they fall under the auspices of the health service director general. According to the Omani National Center for Statistical Information, the catchment population of the SBG was 437,818 in 2019 [14].

### Participants: inclusion and exclusion criteria

Any oral antibiotic prescriptions for outpatients and inpatients of any age issued between January 1 and December 31, 2019 and registered in the Al Shifa system were included.

The following exclusion criteria were applied: antibiotic prescriptions by a general dentist or other specialists in polyclinics; antibiotic prescriptions for tuberculosis, as the treatment options are fixed and standardized; and topical antibiotics, such as ophthalmic ointments and skin creams. Intravenous (IV) antibiotic prescriptions were excluded as the general physicians are not authorized to prescribe IV antibiotics. Patients issued antibiotics for prophylaxis and those who were referred from another PHC institute for antibiotics owing to a lack of availability were also excluded as those prescriptions were not written by the study’s targeted physicians.

### Sampling technique

SBG’s primary health facilities are divided into 3 categories: health centers, extended health centers (polyclinics) and local hospitals. A one stage stratified random sampling technique was used to select the antibiotic prescriptions from six health facilities that were selected randomly. They were Rustaq Polyclinic, Barka Polyclinic, Wadi Bani Ghafir Hospital, Wadi Mistal Hospital, Wudam Health Center, and Wadi Bani Ouf Health Center. The information technology (IT) department that manages the Al Shifa system was contacted to obtain the total count and data of antibiotic prescriptions from the included health facilities. An estimated 37,197 antibiotic prescriptions were issued by general practitioners in SBG during the study period.

Since it was not feasible to work on the high number of prescriptions for the 2019 year, a sample size was calculated with a 95% confidence level. Evidence from the literature indicates that the percentage of inappropriate antibiotic prescriptions in GCC ranges between 30% and 80% [5] It was therefore decided to consider the prevalence to be 50% with a margin error of 4%:

The number of antibiotic prescriptions for each health institute was collected from the IT department. Table 1 shows the proportional distribution for the six selected health institutes. Then, a systematic random sampling technique was used to recruit the antibiotic prescriptions for each health facility. If the prescription were to fail to fulfil the inclusion criteria, then it would be omitted and the following prescription assessed.

**Table 1:** Proportional distribution of antibiotic prescriptions among the selected health institute in SBG

Sn	Institution	No. of Antibiotic prescriptions in 2019	%	*n*
1	Barka Polyclinic	20,465	55.0%	330
2	Rustaq Polyclinic	9176	24.7%	148
3	Wudam Health Center	5607	15.1%	90
4	Wadi Bani Ghafir Hospital	873	2.3%	14
5	Wadi Mistal Hospital	589	1.6%	10
6	Wadi Bani Ouf Health Center	487	1.3%	8
	Total	37,197	100%	600

### Categorization of general physicians’ workload

A primary care physician can reasonably care for 25 patients per day [15], and so the health institute was categorized based on workload according to the following scale: ≤20, 21–25, 26–30, > 30 patients per day [15].

### Categorization of antibiotic prescription appropriateness

The antibiotic prescriptions selected by systematic random sampling for each health institute were reviewed by the primary investigator to verify the diagnosis using the patient’s history, physical examination findings and laboratory investigation results, and by looking for any signs and symptoms that suggested bacterial infection. The Oxford Handbook of Clinical Diagnosis (OHCD) [16] was used as a reference, as it explicitly describes clinical diagnoses and the associated signs and symptoms. Additionally, adult and pediatric out-patient treatment recommendations from the CDC were used as they describe some of the common diagnoses [17, 18] If a record were to show a diagnosis that did not suggest the use of antibiotics, then the patient’s history, examination, and laboratory tests were used to create a second diagnosis, which was worked out by the first author in reference to either the CDC recommendations or OHCD guidelines. The antibiotic prescription would be classified as unnecessary if the history and physical examination were incomplete or not documented and the diagnosis was not suggestive of antibiotic at the same time. Similarly, if the history and physical examination were complete but the diagnosis needed a missing laboratory or radiological finding to confirm the bacterial infection, it was considered an unnecessary prescription. The primary diagnoses of all diseases were followed in accordance with the International Classification of Diseases, Version 10 (ICD-10-CM) [19]. Diagnoses were classified according to physiological system, e.g., respiratory, gastrointestinal, genitourinary or musculoskeletal. An inventory of the antibiotics permitted to be prescribed by general practitioners in Oman is shown in group 1 in Oman guidelines for antibiotic prescription; in order to maintain an appropriate prescribing practice, a number are limited to use by specialists [20].

In addition, the Oman’s MoH approved guidelines for antibiotic prescription and the Oman National Formulary for Ministry of Health Institutions were used as a reference to decide on the appropriateness of the type of antibiotics prescribed [20, 21]. In cases not covered by the latter, the CDC adult and pediatric out-patient treatment recommendations were applied [17, 18]. Antibiotic prescriptions were categorized into appropriate and inappropriate prescriptions. The latter were sub-categorized into 3 groups: (i) unnecessary prescriptions defined as use of antibiotics for diagnoses where antibiotic treatment is not recommended (e.g., viral infections).; (ii) incorrect spectrum of antibiotics defined as the use of antibiotics for diagnosis on a spectrum other than the recommended(e.g., the use of aminoglycosides for gram-positive bacteria); and (iii) unnecessary use of broad-spectrum antibiotics defined as the use of broad spectrum antibiotics in a diagnosis where narrow spectrum is recommended, (e.g., the prescription of cephalosporin instead of penicillin) [3]. The prescription of a systemic antibiotic was regarded as an unnecessary prescription when a topical antibiotic was suggested. If a recommended first-line antibiotic were overlooked and a second-line antimicrobial agent prescribed, this action would be deemed to be inappropriate as a result of unnecessary use of broad-spectrum antibiotics.

### Data collection

The record-based data were obtained from the Al Shifa System. These data were then collected from each institute individually and linked to the antibiotic prescriptions in the Al Shifa system. Permission to log in to the Al Shifa system was approved by the IT department. This allowed the investigators to confirm the history, physical examination and laboratory investigation findings, the latter including the tests typically available in PHC facilities, i.e., full blood count, C-reactive protein titers, and urine and stool analyses, together with the relevant day’s workload details regarding the prescribing doctor.

### Types of variables

Independent variables included: patient’s age, gender, laboratory test status, i.e., tested or not tested; physician’s age, workload (number of patients seen), physician’s language, and institution type.

The dependent variables comprised the appropriateness of antibiotic prescription in accordance with Oman guidelines for antibiotic prescription.

### Data analysis

The collected data were entered in Microsoft Excel. Descriptive analysis was performed by stratifying the prescriptions according to clinical diagnosis, type of antibiotic and health institute workload versus appropriateness. A bivariate analysis was used to determine the association between physician and patient characteristics, and the rate of inappropriate prescription of antibiotics. To account for the confounding influence of other variables, a logistic regression model was applied to the variables that had a p-value of less than 0.25 in bivariate analysis. The Statistical Package for the Social Sciences software, version 23.0 (IBM Corp., Armonk, NY), was used to analyse the data. A p-value of < 0.05 indicate statistical significance.

## Results

600 antibiotic prescriptions were included in the study. These were further categorized based on diagnosis into 9 body system groups (Table 2). The median patient age (interquartile range, Q1-Q3) was 8.3 (3.6–26.9) years, while the median for physician age were 41 (35–48) years. The mean ± standard deviation workload was 34 ± 11 patients per day.

**Table 2:** Distribution of antibiotic prescriptions stratified by clinical diagnosis and appropriateness of use

Diagnosis	Appropriate	Inappropriate			Total
	*N* (%)	Escalated use of extended-spectrum *N* (%)	Incorrect spectrum *N* (%)	Unnecessary *N* (%)	*N* (%)
**Respiratory System Disease**	**29(7.8%)**	**2(0.5%)**	**1(0.3%)**	**340(91.4%)**	**372(62.0%)**
**Upper Respiratory**					
Acute pharyngitis, unspecified	8(3.4%)	0(0%)	0(0%)	225(96.6%)	233(38.8%)
Acute tonsillitis, unspecified	13(12.3%)	2(1.8%)	1(0.9%)	90(84.9%)	106(17.9%)
Acute sinusitis	3(100.0%)	0(0%)	0(0%)	0(0%)	3(0.5%)
Other	0(0%)	0(0%)	0(0%)	4(100.0%)	4(0.7%)
**Lower Respiratory**					
Acute bronchiolitis, unspecified	0(0%)	0(0%)	0(0%)	4(100.0%)	4(0.7%)
Acute bronchitis, unspecified	0(0%)	0(0%)	0(0%)	6(100.0%)	6(1.0%)
Pneumonia	5(83.3%)	0(0%)	0(0%)	1(16.7%)	6(1.0%)
Other	0(0%)	0(0%)	0(0%)	10(100.0%)	10(1.7%)
**Digestive System Disease**	**13(23.2%)**	**0(0%)**	**6(10.7%)**	**37(66.1%)**	**56(9.3%)**
Gastroenteritis of presumed infectious origin	12(28.6%)	0(0%)	6(14.3%)	24(57.1%)	42(7.0%)
Gingivitis	1(10.0%)	0(0%)	0(0%)	9(90.0%)	10(1.7%)
Other	0(0%)	0(0%)	0(0%)	4(100%)	4(0.7%)
**Injury and Poisoning**	**12(21.8%)**	**0(0%)**	**0(0%)**	**43(78.2%)**	**55(9.2%)**
Open wound of the unspecified	7(14.9%)	0(0%)	0(0%)	40(85.1%)	47(7.8%)
Infected wound	5(100.0%)	0(0%)	0(0%)	0(0%)	5(0.8%)
Other	0(0%)	0(0%)	0(0%)	3(100.0%)	3(0.5%)
**Skin and Subcutaneous Disease**	**25(65.8%)**	**2(5.3%)**	**1(2.6%)**	**10(26.3%)**	**38(6.3%)**
Cellulitis, unspecified	9(69.2%)	2(15.4%)	0(0%)	2(15.4%)	13(2.2%)
Cutaneous abscess, furuncle, and carbuncle, unspecified	16(94.1%)	0(0%)	1(5.9%)	0(0%)	17(2.8%)
Other	0(0%)	0(0%)	0(0%)	8(100.0%)	8(1.3%)
**Eye and ENT Disease**	**11(35.5%)**	**0(0%)**	**0(0%)**	**20(64.5%)**	**31(5.2%)**
Otitis media, unspecified	11(73.3%)	0(0%)	0(0%)	4(26.7%)	15(2.5%)
Chalazion	0(0%)	0(0%)	0(0%)	3(100.0%)	3(0.5%)
Other	0(0%)	0(0%)	0(0%)	13(100.0%)	13(2.2%)
**Genitourinary System Disease**	**13(50.0%)**	**8(30.8%)**	**2(7.7%)**	**3(11.5%)**	**26(4.3%)**
Cystitis	13(54.2%)	8(33.3%)	2(8.3%)	1(4.2%)	24(4.0%)
Other	0(0%)	0(0%)	0(0%)	2(100.0%)	2(0.3%)
**Circulatory System Disease**	**7(100.0%)**	**0(0%)**	**0(0%)**	**0(0%)**	**7(1.2%)**
Sepsis	7(100.0%)	0(0%)	0(0%)	0(0%)	**7(1.2%)**
**Musculoskeletal System Disease**	0(0%)	0(0%)	0(0%)	**3(100.0%)**	**3(0.5%)**
Pain in joint	0(0%)	0(0%)	0(0%)	1(100.0%)	1(0.2%)
Low back pain Lumbar region	0(0%)	0(0%)	0(0%)	1(100.0%)	1(0.2%)
Pain in limb	0(0%)	0(0%)	0(0%)	1(100.0%)	1(0.2%)
**Symptoms and Signs Not Elsewhere Classified**	**0(0%)**	**0(0%)**	**0(0%)**	**12(100.0%)**	**12(2.0%)**
Fever, unspecified	0(0%)	0(0%)	0(0%)	6(100.0%)	6(1.0%)
Headache	0(0%)	0(0%)	0(0%)	1(100.0%)	1(0.2%)
Pain, unspecified	0(0%)	0(0%)	0(0%)	3(100.0%)	3(0.5%)
Other	0(0%)	0(0%)	0(0%)	2(100.0%)	2(0.3%)
**Grand Total**	**110(18.3%)**	**12(2.0%)**	**10(1.7%)**	**468(78.0%)**	**600(100.0%)**

81.7% of all antibiotic prescriptions were inappropriately prescribed (Table 2). Respiratory, digestive system diseases, and physical injuries accounted for 62%, 9.3% and 9.2% of antibiotic prescriptions, respectively. However, a smaller number of antibiotics were prescribed for symptoms and signs not classified elsewhere (2%) and musculoskeletal system diseases (0.5%); in which all were inappropriately prescribed. Inappropriate prescription rates for respiratory diseases, injuries and gastrointestinal system diseases were 92.2%, 78.2% and 76.8%, respectively. Inappropriate escalated use of extended-spectrum antibiotics was seen in 33.3% of patients with cystitis; antimicrobial agents from the incorrect spectrum were prescribed in 14.3% and 8.3% cases of gastroenteritis and cystitis, respectively. Antibiotic prescriptions were 100% appropriate when prescribed for acute sinusitis, sepsis and infected wounds, and 94.1% and 83.3% appropriate when administered to patients with cutaneous abscesses and pneumonia, respectively.

Table 3 demonstrates the prescribed antibiotics stratified by the appropriateness of prescription. Amoxicillin and cephalexin accounted for 46.2% and 21.7%, respectively, of all antibiotic prescriptions; 84.4% and 86.2%, respectively, of these were issued unnecessarily. Ciprofloxacin, which was used as a second-line antibiotic, comprised 0.8% of all antibiotic prescriptions. Metronidazole prescriptions formed 15.4% of the incorrect spectrum of antibiotic prescriptions. Appropriate antibiotic prescription was most commonly observed with respect to the use of cloxacillin (41.3%).

**Table 3:** Distribution of antibiotic prescriptions stratified by antibiotic name and appropriateness of use

Antibiotic	Appropriate	Inappropriate			Total
		Escalated use of extended-spectrum N (%)	incorrect spectrum N (%)	Unnecessary N (%)	
Amoxycillin	40(14.4%)	2(0.7%)	0(0%)	235(84.8%)	277(46.2%)
Augmentin	12(26.1%)	1(2.2%)	0(0%)	33(71.7%)	46(7.7%)
PHenoxymethylpenicillin	0(0%)	0(0%)	0(0%)	2(100.0%)	2(0.3%)
Cloxacillin	26(41.3%)	0(0%)	0(0%)	37(58.7%)	63(10.5%)
Cefuroxime	4(21.1%)	5(26.3)	0(0%)	10(52.6%)	19(3.2%)
Cephalexin	15(11.5%)	0(0%)	3(2.3%)	112(86.2%)	130(21.7)
Ciprofloxacin	1(20.0%)	4(80.0%)	0(0%)	0(0%)	5(0.8%)
Erythromycin	0(0%)	0(0%)	0(0%)	18(100.0)	18(3.0%)
Metronidazole	12(30.8%)	0(0%)	6(15.4%)	21(53.8%)	39(6.5%)
Nalidixic acid	0(0%)	0(0%)	1(100.0%)	0(0%)	1(0.2%)
Total	110(18.3%)	12(2.0%)	10(1.7%)	468(78.0%)	600(100.0%)

**Fig. 1:**
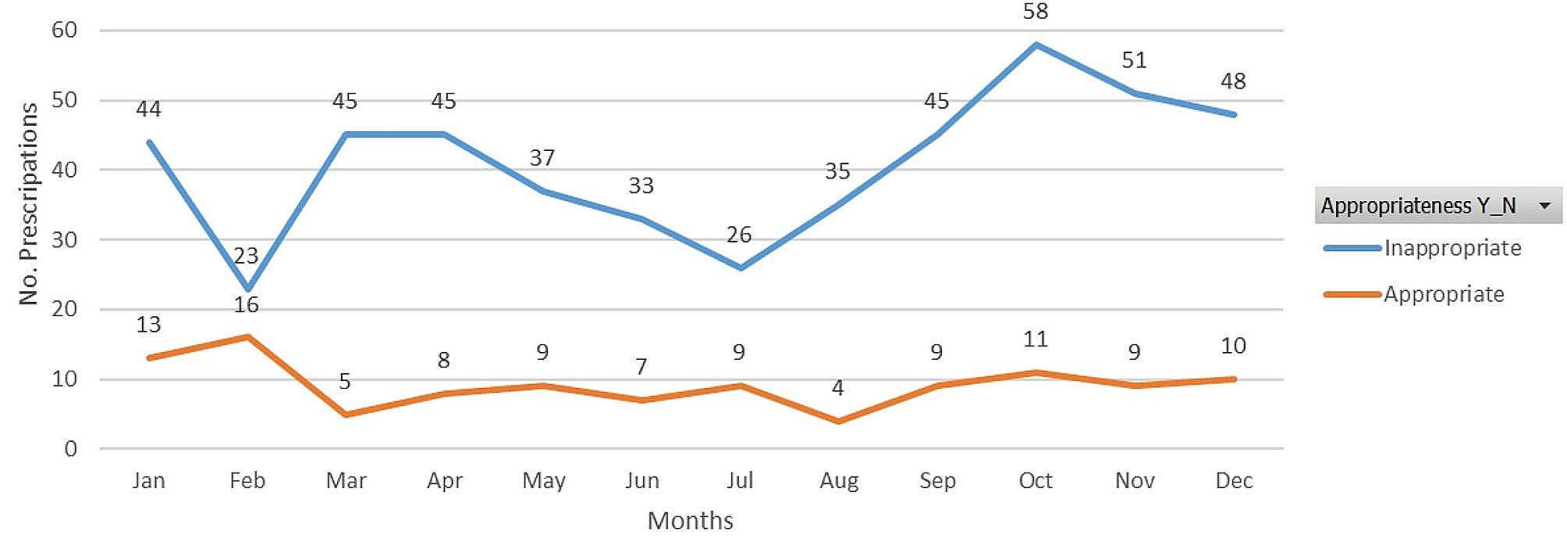
Distribution of antibiotics prescriptions based on appropriateness during 2019 in SBG Oman

The data in Fig. 1 show that inappropriate antibiotic prescriptions gradually decreased from March 2019 to July 2019, at which point they started to increase reaching a peak in October 2019. This was followed by a decline in prescription frequency for the remaining months of the year.

Table 4 demonstrate the bivariate and multivariate analysis of factors associated with the inappropriate prescription of antibiotics. The odds of inappropriate antibiotic prescriptions were (OR = 0.46, 95% CI [0.26, 0.82]) and (OR = 0.33, 95% CI [0.14, 0.78]) among patients aged 18–50 years and > 50 years, respectively, compared to those aged 5 years. The odds of inappropriate prescription of antibiotics were (OR = 0.22, 95% CI [0.12, 0.39]) among patients who were tested by laboratory investigations. The odds of inappropriate prescription of antibiotics among physicians who work in a health center, compared to those who work in a polyclinic and those who are Arabic-speaking physicians compared to non-Arabic speaking physicians, were (OR = 0.44, 95% CI [0.24, 0.79]) and (OR = 1.63, 95% CI [1.001, 2.643]), respectively.

**Table 4:** A bivariate and multivariate analysis of factors associated with the appropriateness of use

Characteristics		Appropriate	Inappropriate			Total	Crude OR (95% CI)	*P*-value	Adjusted OR (95% CI)
		*N* (%)	Escalated use of extended-spectrum *N* (%)	Incorrect spectrum *N* (%)	Unnecessary *N* (%)				
Total		110(18.3%)	12(2.0%)	10(1.7%)	468(78.0%)	600 (100.0%)			
Patient Characteristics									
Sex	Female	51(17.7%)	10(3.5)	4(1.7%)	223(77.4%)	288(48%)	Ref		*Not included
	Male	59(18.9%)	2(0.6%)	6(1.9%)	245(78.5%)	312(52%)	0.92 (0.61, 1.39)	0.70	Not included
Age Group (Years)	≤ 5	29 (13.7%)	1(0.5%)	1(0.5%)	180(85.3)	211(35.2%)	Ref		Ref
	6–17	22(12.4%)	2(1.1%)	3(1.7%)	150(84.7%)	177(29.5%)	1.12(0.62, 2.03)	0.70	1.18(0.63, 2.22)
	18–50	46(26.4%)	8(4.6%)	6(3.4)	114(65.5%)	174(29.0%)	0.44(0.26, 0.74)	< 0.01	0.46(0.26, 0.82)
	> 50	13(34.2%)	1(2.6%)	0(0.0%)	24(63.2%)	38(6.3%)	0.31(0.14, 0.67)	< 0.01	0.33 (0.14, 0.78)
Lab test	Not Tested	79(14.8%)	3(0.6%)	2(0.4%)	451(84.3%)	535(89.2%)	Ref		Ref
	Tested	31(47.7%)	9(13.8%)	8(12.3%)	17(26.2%)	65(10.8%)	0.19(0.11, 0.33)	< 0.01	0.22(0.12, 0.39)
Doctor Characteristics									
Sex	Female	59(19.0%)	8(2.6%)	4(1.3%)	240(77.2)	311(51.8%)	Ref		Not included
	Male	51(17.6%)	4(1.4%)	6(2.1%)	228(78.9%)	289(48.2%)	1.09(0.72, 1.65)	0.68	Not included
Age Group (Years)	25–31	6(10.9%)	0(0)	1(1.8%)	48(87.3%)	55(9.2%)	Ref		Ref
	32–38	50(24.5%)	4(2.0%)	4(2.0%)	146(71.6%)	204(34.0%)	0.38(0.15–0.93)	0.03	0.49(0.18, 1.30)
	39–75	54(15.8%)	8(2.3%)	5(1.5%)	274(80.4%)	341(56.8%)	0.65(0.27–1.60)	0.34	0.98(0.36, 2.64)
Workload	<=20	11(16.2%)	3(4.4%)	4(5.9%)	50(73.5%)	68(11.3%)	1.09(0.55, 2.20)	0.80	0.16(1.79, 4.07)
	21–25	11(17.7%)	0(0%)	2(3.2%)	49(79.0%)	62(10.3%)	0.98(0.49, 1.98)	0.95	1.11(0.52, 2.35)
	26–30	20(25.0%)	2(2.5%)	0(0%)	58(72.5%)	80(13.3%)	0.63(0.36, 1.12)	0.12	0.60(0.32, 1.10)
	> 30	68(17.4%)	7(1.8%)	4(1.0%)	311(79.7%)	390(65.0%)	Ref		Ref
Institute Type	Polyclinic	81(16.9%)	12(2.5%)	7(1.5%)	378(79.1%)	478(79.7%)	Ref		Ref
	Health Center	25(25.5%)	0(0.0%)	3(3.1%)	70(71.4%)	98(16.3%)	0.60(0.36, 0.995)	0.046	0.44(0.24, 0.79)
	Local Hospital	4(16.7%)	0(0.0%)	0(0.0%)	20(83.3%)	24(4.0%)	1.02(0.34, 3.06)	0.97	0.32(0.09, 1.14)
Doctor Language	Non-Arabic	52(20.6%)	5(2.0%)	5(2.0%)	191(75.5%)	253(42.2%)	Ref		Ref
	Arabic	58(16.7%)	58(16.7)	7(2.0%)	5(1.4%)	347(57.8%)	1.29(0.85, 1.95)	0.23	1.63(1.001, 2.643)

*Not included: as the p-value of crude odd ratio was more than 0.25 we didn’t include it in multivariate analysis

Measures of goodness of fit: Omnibus χ2 (12) = 68.54, *p* < 0.001, R2 = 0.108 (Cox & Snell), 0.176 (Negelkerke), Hosmer and Lemeshow test, A non-significant chi-square *P* = 0.985

## Discussion

This study was conducted to evaluate the appropriateness of antibiotic prescriptions issued by general practitioners in PHC in the SBG of Oman. The most frequently arising inappropriate prescriptions were written in the context of respiratory diagnoses, and involved the antimicrobial agent, amoxicillin. Associations were identified between the inappropriate prescription of antibiotics and the patient’s age and testing status, and the physician age, working institute and language spoken.

A high level, i.e. 81.7%, of inappropriate antibiotic prescriptions was estimated during the course of this research. Studies in Qatar, Ecuador, Jordan and Italy reported the degrees of inappropriate prescription of antibiotics among respiratory cases to be 45%, 90.25%, 72.2% and 66.5%, respectively [22]. In the current study, just over 60% of antibiotic prescriptions were related to respiratory disease, of which 92.2% were inappropriately prescribed. Acute pharyngitis and acute tonsillitis were the most common diagnoses; of which, most were issued with unnecessary prescriptions. Indeed, 90% of pharyngitis and tonsillitis cases are caused by viral infections; only 5–10% are caused by bacterial infections that require antibiotics [18].

Amoxycillin was the most prescribed antibiotic, but the majority of the prescriptions were judged to be unnecessary. Studies in Spain and Italy reported the use of amoxicillin in 36% and 56% of cases, respectively, particularly in patients with acute pharyngitis [23] This can be explained by the fact that amoxicillin is one of the most affordable first-line broad-spectrum antibiotics; a course costs 1.9 US$ [24].

A seasonal variation in inappropriate antibiotic prescription was evident, reaching a peak in October, 2019. An expectation was that the frequency of inappropriate antibiotic prescriptions would increase in the winter months, when there is a higher incidence of viral infections of the respiratory tract. A similar trend was seen in primary care in both Switzerland and England between September and December, when the maximum number of antibiotics was prescribed [8, 25].

The current study showed that patients over 18 years of age were less likely to get an inappropriate antibiotic prescription compared to those younger than 5 years, which corresponds with studies carried out in Ethiopia, Sudan and Portugal [26] In many situations, patients in the younger age group present with different non-specific symptoms, and it may be challenging to reach a diagnosis without laboratory investigations. A survey conducted among pediatricians found that 96% of parents insist that their children should have antibiotics, and fail to be satisfied by analgesic medication alone [27] This reflects the need for more awareness among the general public about the appropriate prescription of antibiotics and for additional regulations for medical practices [27].

Patients in the current study who had undergone laboratory tests were less likely to be treated inappropriately. It was found that the testing practice maximized the appropriateness of the antibiotic prescription, which led to the minimal prescription of empirical antibiotics and stronger adherence to guideline recommendations [28] The unavailability of rapid diagnostic tests, antibiotic susceptibility data for most common bacteria, and the weak implementation of an antibiotic stewardship program have been demonstrated to be linked to the prescription of antibiotics from an incorrect spectrum [29].

Our study found no significant association between physicians’ ages and inappropriate antibiotic prescriptions. This is different from a study in China, where primary care physicians who were older than 32 years were less likely to prescribe antibiotics inappropriately [3]. Physicians working in health centers were less likely to prescribe antibiotics inappropriately compared to those working in polyclinics. This observation could be explained by the limited stock of antibiotics available in health centers which may result in strict rules and regulations being applied to control the prescription and dispensation of antibiotics in this context. Such control leads to a reduction in the correct spectrum and increased prescription of broad-spectrum antibiotics [9, 10]. The Arabic-speaking doctors tended to prescribe antibiotics inappropriately compared to non-Arabic speaking physicians. Patients may exert pressure on Arabic-speaking doctors, which could increase the prescription of inappropriate antibiotics [30] Furthermore, the lack of a language barrier infers that many Arabic speaking doctors could have a better relationship with their patients and would therefore find it hard to refuse their requests for antibiotics [31].

This study would encourage primary health care facilities to consider improving their testing capacity, in order to help reach the correct diagnosis and select the corresponding recommended antibiotic. Undoubtedly, it remains important to educate physicians, particularly with respect to the use of antibiotic stewardship programs, as well as provide community health education for parents.

### Limitations

The current study has some limitations. Firstly, the diagnosis selection could vary from one physician to another; some of the physicians did not use the appropriate ICD-10 diagnosis. However, to minimize this limitation, the second diagnosis was worked out based on the findings from the history, physical examination and laboratory tests. Furthermore, owing to the presence of specialty clinics; local hospitals and polyclinics have an extended list of antibiotics which is not available in health centers. Thus, the general practitioner’s decision might be affected by the availability of antibiotics or by the advice of specialists. The study was conducted in governmental health centers, and so the findings cannot necessarily be generalized to general practitioners in the private sector. Missing data related to patient history and physical examination findings were observed in less than 5% of records; an assumption of inappropriate prescription was made in such instances, which was thought to be most likely. The duration and doses of antibiotics are important elements to evaluate the appropriate prescription of antibiotics, which could be carried out in future studies.

## Conclusion

The inappropriate antibiotic prescription was frequently observed in mild respiratory infections and associated with some patient and physician characteristics. Improvement of the appropriateness of antibiotic prescriptions could be achieved by the implementation of physician education initiatives, awareness campaigns for the community, and enhancement of testing capacities.

### Electronic supplementary material

Below is the link to the electronic supplementary material.

Supplementary Material 1

## Data Availability

All data from the Al shift system on antibiotic prescriptions, related patients, and prescribing physicians that support the findings of this study are included in this paper and its Supplementary Information files.

## Abbreviations

AMR: Antimicrobial resistance
DDD: Defined daily doses
SQUH: Sultan Qaboos University Hospital
PHC: Primary healthcare
SBG: South Batinah Governorate
GCC: Gulf Cooperation Countries
MoH: Ministry of Health
IV: Intravenous
IT: Information technology
OHCD: Oxford Handbook of Clinical Diagnosis
CDC: Centers for Disease Control and Prevention
